# Recognition of Knee Osteoarthritis (KOA) Using YOLOv2 and Classification Based on Convolutional Neural Network

**DOI:** 10.3390/life12081126

**Published:** 2022-07-27

**Authors:** Usman Yunus, Javeria Amin, Muhammad Sharif, Mussarat Yasmin, Seifedine Kadry, Sujatha Krishnamoorthy

**Affiliations:** 1 Department of Computer Science, COMSATS University Islamabad, Wah Campus, Wah Cantt 47010, Pakistan; usmanyunus64@gmail.com (U.Y.); muhammadsharifmalik@yahoo.com (M.S.); mussaratabdullah@gmail.com (M.Y.); 2 Department of Computer Science, University of Wah, Wah Cantt 47010, Pakistan; javeria.amin@uow.edu.pk; 3 Department of Applied Data Science, Noroff University College, 4612 Kristiansand, Norway; skadry@gmail.com; 4 Zhejiang Bioinformatics International Science and Technology Cooperation Center, Wenzhou-Kean University, Wenzhou 325060, China; 5 Wenzhou Municipal Key Lab of Applied Biomedical and Biopharmaceutical Informatics, Wenzhou-Kean University, Wenzhou 325060, China

**Keywords:** knee osteoarthritis (KOA), handcrafted features, KL grading, features fusion, classification, localization

## Abstract

Knee osteoarthritis (KOA) is one of the deadliest forms of arthritis. If not treated at an early stage, it may lead to knee replacement. That is why early diagnosis of KOA is necessary for better treatment. Manually KOA detection is a time-consuming and error-prone task. Computerized methods play a vital role in accurate and speedy detection. Therefore, the classification and localization of the KOA method are proposed in this work using radiographic images. The two-dimensional radiograph images are converted into three-dimensional and LBP features are extracted having the dimension of N × 59 out of which the best features of N × 55 are selected using PCA. The deep features are also extracted using Alex-Net and Dark-net-53 with the dimensions of N × 1024 and N × 4096, respectively, where N represents the number of images. Then, N × 1000 features are selected individually from both models using PCA. Finally, the extracted features are fused serially with the dimension of N × 2055 and passed to the classifiers on a 10-fold cross-validation that provides an accuracy of 90.6% for the classification of KOA grades. The localization model is proposed with the combination of an open exchange neural network (ONNX) and YOLOv2 that is trained on the selected hyper-parameters. The proposed model provides 0.98 mAP for the localization of classified images. The experimental analysis proves that the presented framework provides better results as compared to existing works.

## 1. Introduction

In the world, around 30% of people over the age of 60 have OA, which is the main cause of impairment in the elderly. Over 250 million patients are suffering from this disease globally [1]. Primary KOA symptoms are pain, stiffness, decreased range of joint motion, and malfunctioning gait that ultimately increases the progression rate of the disease [2]. These indications affect the individuals’ functional independence and degrade their life quality. The Kellgren–Lawrence (KL) grading system is used as a gold standard for assessments of KOA radiographs. The KL grading system classifies KOA into 0–4 grades, where grade 0 represents healthy with no symptoms of KOA while grade 4 presents a severe stage [3]. The KL grading system is commonly used clinically for KOA diagnosis which is time consuming and needs skilled experts. For accurate KL grading evaluation, two skilled experts are required that could independently process the radiographs without considering other input data [4]. The computerized system is developed for the automated labeling of KOA severity using a deep siamese convolution neural network. This method is trained on the MOST dataset in which 3000 testing subjects are selected randomly out of 5960 hence providing an average accuracy of 66.7% and 0.83 co-efficient of kappa [5]. Sobel horizontal gradient with SVM classifier is used for the diagnosis of knee abnormality using X-ray radiographs [6]. The automated KOA method is presented and tested on 94 images of radiographs that provides a 72.61% precision rate. Due to the poor contrast and variable locations of knee gaps, detecting KOA is a difficult process [7].

The method for the classification and localization of knee OA is proposed here to address these issues. The core contribution is as:

For accurate classification, KOA images are converted into three channels. After conversion, LBP and deep features are derived using Darknet-53 and Alex-Net and fused serially to select the best features by PCA that are input to the classifiers for KOA grades. The classified images are supplied into the proposed localization model, which extracts features from the ONNX model and feeds them into the YOLOv2 detector. The optimal hyper-parameters are used for model training to accurately localize the infected knee region.

The remaining article is organized as: Section 2 gives related work, the proposed model is explained in Section 3, results and discussion are written in Section 4, and Section 5 gives a conclusion.

## 2. Related Work

KOA is a complex peripheral joint disease with many risk factors that contribute to significant loss of control, weakness, and rigidity [8]. Its severity level is computed manually through the KL grading system, but it takes time and can lead to misclassification. There has been plenty of work carried out in the area of KOA imaging to identify and classify knee diseases. In image processing, feature extraction is an effective step for image representation [9,10,11,12,13,14,15,16,17,18,19,20,21,22,23,24,25,26,27,28,29,30,31,32,33,34,35,36,37,38,39,40,41,42,43,44,45,46,47,48]. For the recognition of diseases, feature extraction is very helpful to machine learning (ML) algorithms. Many researchers used handcrafted features for KOA classification [49]. A new computer-based approach is proposed for segmenting knee menisci in MR images with the help of handcrafted features named HOG and LBP in which they used the variant of histogram HOG-UoCTTI. The ratio of overlap area is calculated by the Dice similarity formula to select 31 and 58 features of LBP and HOG, respectively. In knee MR, 45 slices are under evaluation, so after random sub-sampling, the size of the feature matrix is 7000 × 837 for each image. These features are selected by using PCA and achieved 82% Dice similarity [50]. Saygili et al. presented automated detection of knee menisci from MR images. These images are obtained from the OAI dataset such that 75% of these are taken for training while 25% are for testing. Features are extracted with the HOG method for both testing and training processes. To find the correlation between different patches, the regression approach is used in the training process [51]. Mahrukh et al. used a HOG-based template matching automated technique for required region extraction named tibiofemoral in knee radiographs [52]. Their methodology achieved an accuracy of 96.10% with an average mean rate of 88.26%, which exceeds current strength approaches such as fuzzy-c means and deep models [53]. A three-dimensional deformation technique for homogeneity in the knee was developed and evaluated. D. Kim et al. demonstrated that the current issue could be solved depending on the histogram. Explanatory results have shown 95% Dice similarity, 93% sensitivity, and 99% specificity [54]. An adaptive segmentation method is presented for selecting ROI by using different handcrafted methods and improving the classification process. After the pre-processing of raw data images from OAI and MOST databases, they chose ROI to calculate texture descriptors. In their studies, they used both (rectangular ROI and adaptive ROI) techniques [55]. Fractal dimension (FD) [56], local binary pattern (LBP), Haralick features [57], Shannon entropy [57], and HOG have been analyzed and compared. Their proposed method achieved an improvement of 9% in AUC as compared to commonly used ROI, and LBP provided the best performance in all features [55]. In the area of ML, deep learning (DL) has gained more interest in recent years [55]. DL methods are more precise as compared to the approaches focusing on handcrafted features. In medical imaging, several models have been developed such as Alexnet, VGG19 [58], Darknet [59], etc., for the extraction of features. Kevin et al. developed a model for OA diagnosis and total knee replacement by using DL model Resnet-34 [60] which has 34 layers. They trained their model on OAI and WOMAC + OA outcome scores [61], jointly predicted KL grade and TKR on the same model, and achieved a higher AUC of 87% as compared to the previous [62]. B. Zhang et al. developed a model to automatically diagnose KOA. They applied a modified residual neural network by changing the kernel size of the average pooling layer for the detection of a knee joint and then combined it with convolutional (BAM) to achieve the state of art performance from previous methods [63]. For the assessment of tumors in knee bones, H.J. Yang et al. provided an effective DL model. A combination of supervised and unsupervised techniques was used to recognize significant patterns in the identification of prevalent and anomalous bones and also to identify bone tumors. The results indicated that the model performance is better than the existing remarkable models [64]. Vishwanath et al. used an MR high-resolution algorithm with new full 3D CNN and a multi-class loss functionality to develop a segmentation of knee cartilage and achieved better performance on publicly available MICCAI SKI10 dataset. They have also applied their proposed methodology to a similar MR and enhanced segmentation accuracy [65]. In another work, the researchers developed a technique for automatic classification of knee radiography severity. They used the DenseNet CNN model to predict KL grade which has 169 layers [66].

## 3. Proposed Methodology

**Figure 1:**
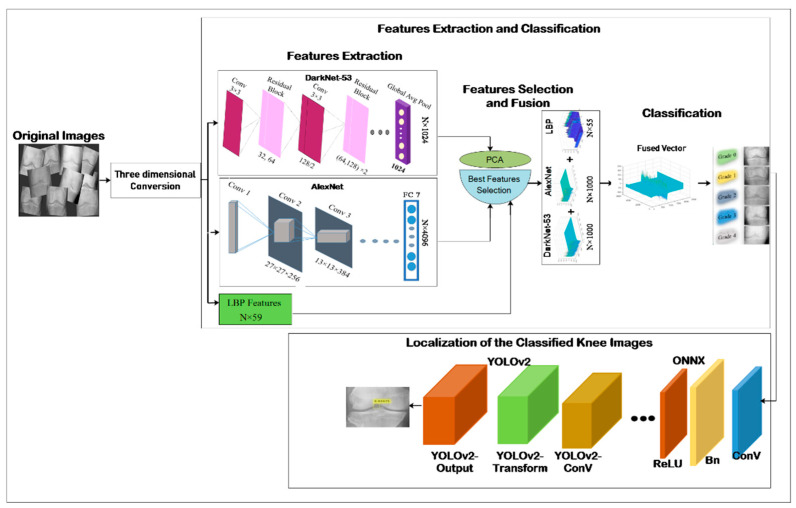
The architecture of the proposed methodology.

This section describes the classification of the KOA method for tackling current limitations and addressing the challenges mentioned above. In this method, deep and LBP features are extracted after which the best features are selected using PCA for classifying different grades of KOA. Then classified images are localized using the YOLOv2-ONNX model. The overall scenario is presented in Figure 1.

### 3.1. Local Binary Pattern (LBP)

LBP [67] is established on the gray level structure of an image and extracts texture features from an image. It works in a form of a 3 × 3 window slider over an image. The center pixel of an image is a threshold value to its neighboring pixels. Each pixel is compared around the window with eight different pixels such that 28 = 256 various patterns for the selected region can be achieved from an image.

**Figure 2:**
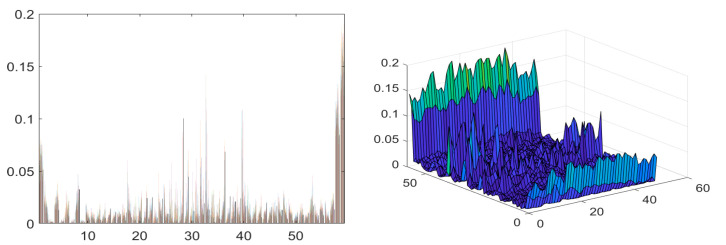
Graphical representation of LBP features.

Figure 2 shows LBP features of dimension N × 59. The LBP operator is given by Equation (1).

(1) $$\xi_{\left(P,R\right)}=\overset{P-1}{\underset{k=0}{\sum }}2^{k}s\left(X_{k}-X_{c}\right)$$

where s denotes the operator that retains sign of differences defined by:

$$s\left(X_{k}-X_{c}\right)=\{\begin{array}{c} 1\;if\;X_{k}-X_{c}\geq 0\\ 0\;if\;X_{k}-X_{c}<0 \end{array}$$

where $X_{c}$ denotes center pixel value, P symbolizes neighboring pixels of $X_{c}$, R represents the radius of the window, and $X_{k}$ denotes pixel intensity values in the neighborhood. The size of the feature vector is N × 59. Once these features are extracted, N × 55 best features are selected by using PCA.

### 3.2. Deep Feature Extraction

CNN is a DL algorithm used for the extraction of important image information and can differentiate various objects from one another. It works in the form of layers named convolution, pooling, and ReLU. Our dataset is on a large scale; hence, CNN is very helpful for feature extraction in image classification. Therefore, features are derived from Alex-net and Darknet-53 models. The Alexnet [68] model consists of 25 layers including five convolutional and three fully-connected (FC6, FC7, and FC8), ReLU (6), drop (5), pooling (5), softmax, and classification. The features are derived from the FC7 layer of the Alexnet model with the dimension of N × 4096. The pre-trained DarkNet53 [69] with the dimensions of 1 × 1 and 3 × 3 has a 53-layer deep model. This model contains 184 layers in which 1 input, 53 Conv, 52 batch-norm, 52 leaky-ReLU, 23 addition, 1 softmax, 1 classification, and 1 average global pooling are included. Features are derived from the pool average layer named avg1 for the activation process to get a vector size of N × 1024 features.

### 3.3. Feature Fusion

**Figure 3:**
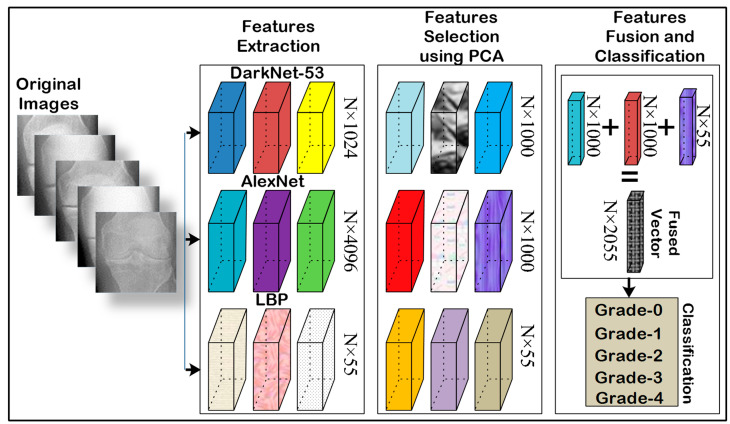
Overview of feature extraction, selection, fusion, and classification.

This step fuses handcrafted and deep features with the dimension N × 2055 for the classification of KOA because 1000 features are selected from Alexnet, and 1000 features from Darknet-53 as well as 55 features from LBP by using PCA [70]. PCA reduced the dimension of larger vectors into smaller ones by keeping its actual information. Figure 3 shows the fusion process of handcrafted and CNN features.

In Figure 3, the feature vector dimension is N × 4096, N × 1025, and LBP descriptor with N × 59 in which N × 1000 deep, and LBPN × 55 features are selected using PCA. Finally, these extracted features are fused with the dimension of N × 2055.

ICA is used to optimize the statistics of high order like kurtosis. PCA is used to optimize the covariance matrix that denotes second-order statistics [68,71]. ICA searches independent components, while PCA searches un-correlated components. The final vector of the fusion process is mathematically defined in Equation (2).

(2) $$\Xi_{\mathrm{fv}}\left(i\right)=\left(\begin{array}{c} \xi_{\left(P,R\right)}{\left(i\right)}_{M\times N}\\ \xi_{\mathrm{av}}{\left(i\right)}_{M\times N}\\ \xi_{\mathrm{dv}}{\left(i\right)}_{M\times N} \end{array}\right)$$

In the above equation, $\xi_{\left(P,R\right)}\left(i\right)$ denotes LBP feature vector, $\xi_{\mathrm{fv}}$(i) is the final vector after the fusion process, $\xi_{\mathrm{av}}$(i) and $\xi_{\mathrm{dv}}$(i) are features vectors of Alexnet and Darknet-53, respectively, while M × N represents the dimensions of these vectors. The SVM [72], KNN [73], and Ensemble classifiers with different kernels are used for classification. To choose the best features, an experiment is conducted using ICA and PCA as mentioned in Table 1.

In this experiment, high accuracy was achieved using PCA as compared to ICA. Therefore, PCA is selected for further experimentation. 

### 3.4. Localization of Knee Osteoarthritis by Using YOLOv2 with the ONNX Model

YOLO-v2 delivers higher efficiency for object detection in terms of accuracy and speed [51]. Extraction of features and location steps are performed by using YOLO-v2 in a single unit. The proposed model YOLO-v2ONNX has 31 layers designed by using YOLO-v2 with the pre-trained architecture of the ONNX [52,53,54] model for the detection of KOA. ONNX model is a multiple output network in which 35 layers are present, but this work used only 24 layers for the preparation of the proposed model as (i) input layer, (ii) 2 element-wise Affine layers, (iii) 4 convolutional layers, (iv) 4 BN layers, (v) 3 max-pooling layers, and (vi) 4 activation layers. These layers are passed to YOLO-v2 which has 3 convolutional layers, 2 BN layers, and 2 ReLUlayers that are serially linked and accompanied by YOLO-v2 transformation and YOLO-v2 output to accurately detect the location in an input image with the class labels of infected regions. 

YOLO-v2ONNX model detects class labels by using anchor boxes. Three major attributes are defined as (a) IoU (b) Offset, and (c) class probability for the prediction of anchor boxes. IoU predicts objects score across each anchor box, the position of the anchor box is defined by an offset, and class probability is measured to calculate relevant class labels allocated to the corresponding anchor boxes. 

The object detector YOLO-v2 improves mean square error (MSE) loss between expected and ground truth bounding boxes. The proposed model is trained on three types of losses to reduce MSE: (a) localization loss in which error is measured between ground truth, and bounding box and parameters for measuring the localization loss as follows.

$$W_{1}\overset{g^{2}}{\underset{k=0}{\sum }}\overset{d}{\underset{l=0}{\sum }}1_{\begin{array}{c} KOA\\ kl \end{array}}\left[{\left(a_{k}-{\hat{a}}_{k}\right)}^{2}+{\left(b_{k}-{\hat{b}}_{k}\right)}^{2}\right]+$$

$$W_{1}\overset{g^{2}}{\underset{k=0}{\sum }}\overset{d}{\underset{l=0}{\sum }}1_{\begin{array}{c} KOA\\ kl \end{array}}\left[{\left({\sqrt{w}}_{k}-{\hat{\sqrt{w}}}_{k}\right)}^{2}+{\left({\sqrt{h}}_{k}-{\hat{\sqrt{h}}}_{k}\right)}^{2}\right]$$

$$+W_{2}\overset{g^{2}}{\underset{k=0}{\sum }}\overset{d}{\underset{l=0}{\sum }}1_{\begin{array}{c} KOA\\ kl \end{array}}{\left(s_{k}-{\hat{s}}_{k}\right)}^{2}+$$

$$W_{3}\overset{g^{2}}{\underset{k=0}{\sum }}\overset{d}{\underset{l=0}{\sum }}1_{\begin{array}{c} noKOA\\ kl \end{array}}{\left(s_{k}-{\hat{s}}_{k}\right)}^{2}$$

$$+W_{4}\overset{g^{2}}{\underset{k=0}{\sum }}1_{\begin{array}{c} KOA\\ k \end{array}}\underset{c\in classes}{\sum }{\left(p_{k}\left(c\right)-{\hat{p}}_{k}\left(c\right)\right)}^{2}$$

Here g denotes grid cells, d shows bounding boxes size, $1_{\begin{array}{c} KOA\\ kl \end{array}}$ = 1 if 1 bounding box is responsible for detecting the object in grid cell k otherwise it is considered 0, $1_{\begin{array}{c} noKOA\\ kl \end{array}}$ = 1 if there is no object detected in 1 bounding box, $1_{\begin{array}{c} KOA\\ k \end{array}}$ = 1 if the object is located otherwise it is considered 0. ($a_{k}$,$b_{k}$) and (${\hat{a}}_{k}$,${\hat{b}}_{k}$) represent the center point of l bounding box and ground truth in grid cell k, while (${\sqrt{w}}_{k}$,${\sqrt{h}}_{k}$) and (${\hat{\sqrt{w}}}_{k}$,${\hat{\sqrt{h}}}_{k}$) denote width and height, and weight of localization loss is denoted by $W_{1}$. The second step is confidence loss. The error of confidence score is measured when the object is detected. When there is no object detected in the l bounding box of grid cell k then the error of confidence score is measured. The parameters for measuring the confidence loss are ($s_{k}$,${\hat{s}}_{k}$) representing the confidence score of the l bounding box and ground truth in grid cell k whereas ($W_{2}$,$W_{3})$ are the weights of confidence score error if the object is detected or not. The last step of the loss function of YOLOv2 called classification loss is used to compute the squared error between the probabilities of each class from which the object is detected in grid cell k of the l bounding box. The $p_{k}\left(c\right)$ and ${\hat{p}}_{k}\left(c\right)$ are the estimated and actual probabilities of conditional class for object class c in grid cell k, and $W_{4}$ represents the classification error weight. With the increase in the value of $W_{4}$, the weightage of classification loss also increases.

## 4. Results and Discussion

In this work, the knee joints dataset is publicly accessible [74] which includes training 2139 images and testing 1656 images. The dataset is in 2 channels, so it is converted into 3 channels (RGB) because deep models accept 3 channel images. This work is implemented on MATLAB-2020 Ra, a Windows operating system with 2070 RTX-GPU.

### 4.1. Experiment #1 (Grades of KOAClassification)

**Figure 4:**
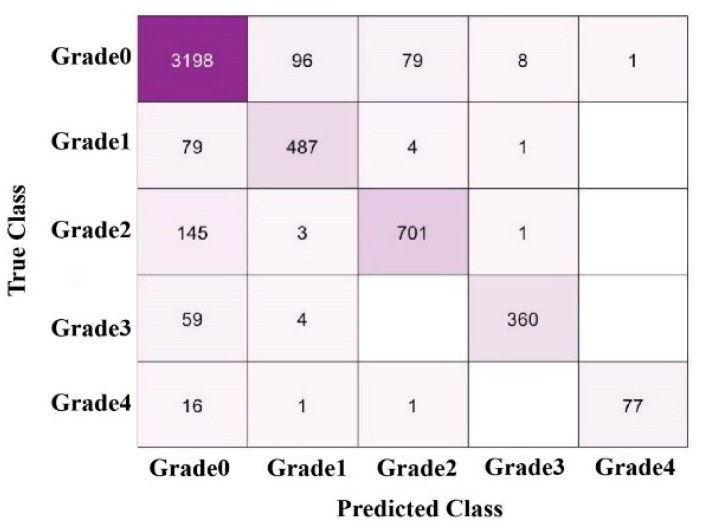
Multi-class confusion matrix.

In this experiment, SVM, KNN, and Ensemble classifiers are used to classify KOA grades into Grade-0 to Grade-4 as manifested in Figure 4.

The sensitivity of 85% is achieved in Grade-I in which 487 true positive and 4179 false negative values are included. As shown in Table 2, a 10-fold cross-validation is used for classification.

In Table 2, overall accuracy obtained on collective KOA grades and individual grades is presented including 90.6% on Fine KNN, 77.9% on SVM, and 89.4% on Ensemble KNN. Maximum precision of 0.97 is attained using SVM on Grade (0), 0.85 on Grade (1,3) based on Ensemble KNN, 0.82 on Grade (2) using Ensemble KNN, and 0.82 on Grade (4) using SVM. The classification results comparison is mentioned in Table 3. 

In Table 3, deep siamese CNN provided 66.7% accuracy. This method needs improvement to increase the detection accuracy [14]. Chen et al. developed a model to automatically measure KOA severity from knee radiographs and provided an accuracy of 69.7% [10]. B. Zhang et al. presented a technique to automatically diagnose KOA. They applied a modified residual neural network by changing the kernel size for the detection of the knee joint and then combined it with convolutional (BAM) to achieve multi-class accuracy of 74.8%. This method also needs to improve the classification accuracy [43]. Kondal et al. [55] used two datasets, one from OAI, which has 4447 DICOM format images with their KL grades for training, and the second dataset is from an Indian private hospital having 1043 knee radiographs. However, they did not obtain high-performance results on this target dataset. They showed average (precision, recall, and F1-score) when their model is evaluated on the OAI dataset. The ensemble fuzzy features selection method is used based on the embedded, wrapper, and filter method with a random forest classifier for the classification of knee grades. This method provides 73.35% accuracy [74]. ResNet-18 and ResNet-34 are used with convolutional attention blocks for the prediction of KL grades. This method achieved 74.81% accuracy [77]. 

After experimentation, we achieved maximum accuracy of 90.6% while the previous maximum accuracy was 84%. Still, there is a gap in this domain due to the complex structure of knee radiographs. Therefore, more novel methods are required to fill this research gap. 

### 4.2. Experiment#2 Localization of Knee Osteoarthritis

**Figure 5:**
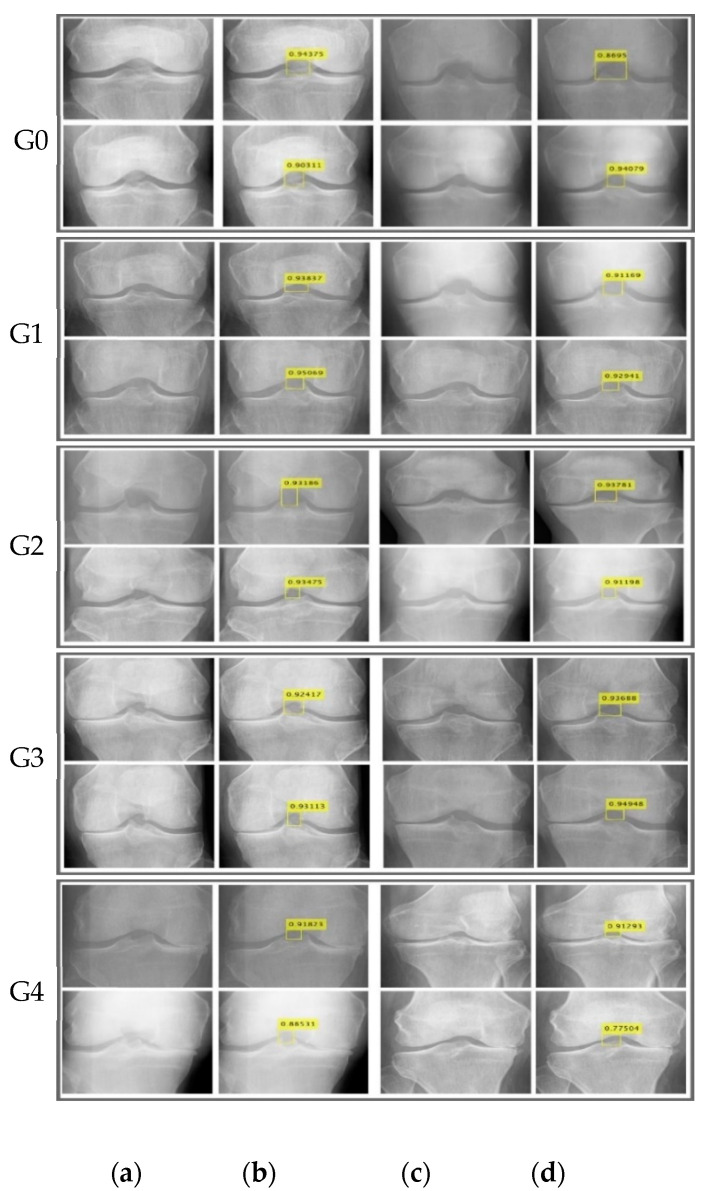
KOA localization results (**a**,**c**) original KOA slices (**b**,**d**) predicted scores (where G denotes grades).

This experiment localized classified images using the proposed localization model into different grades (Grade 0 to Grade 4) of KOA with maximum confidence scores as shown in Figure 5.

Table 4 presents YOLOv2-ONNX model configuration parameters chosen after rigorous testing.

Table 5 shows the outcomes of the proposed localization model in terms of mIoU and mAP.

In Table 5, the existing method [78] provided an IoU of 0.95. In the literature, no method exists for the localization of KOA images. 

## 5. Conclusions

Precise and accurate identification and classification of KOA is a challenging task. The similarity between different KL grades makes it more complex. Its severity level is computed manually through the KL grading system, but it takes time and can lead to misclassification. Automated grading of KOA severity can provide reliable results in a short period. However, various forms of KOA must be handled more carefully. In addition, robust features and efficient classifiers have an immense effect on the efficiency of the diagnosis method. In this study, a new technique is developed for OA detection using radiographic images. The proposed model includes (a) pre-processed original dataset, (b) extraction of handcrafted features, (c) extraction of deep features from pre-trained CNN models, (d) PCA model for the best selection of features, (e) feature fusion, (f) classification, and (g) localization of classified images using the YOLO-v2ONNX model. The proposed technique achieved a precision rate of 0.95 on Grade-0, 0.85 on Grade-1, 0.82 on Grade-2, 0.85 on Grade-3, and 0.81 on Grade-4 with the Ensemble KNN classifier. For the localization of KOA, the YOLO-v2ONNX model is developed by using the ONNX model as the backbone of YOLO-v2 and achieved 0.96 IOU and 0.98 mAP on classified images.

## Figures and Tables

**Table 1:** Experiment for features selection method.

Features Selection Methods	Accuracy
ICA	0.87
PCA	0.90

**Table 2:** Classification outcomes utilizing 10-fold cross-validation.

Classifiers	G0	G1	G2	G3	G4	Accuracy% (ACC)	Precision% (Pre)	Sensitivity% (SE)	F1 Score% (F1)
SVM	✓					77.9	0.97	0.89	0.93
		✓					0.73	0.87	0.80
			✓				0.75	0.90	0.82
				✓			0.81	0.96	0.88
					✓		0.82	0.93	0.87
Fine KNN	✓					90.6	0.97	0.89	0.93
		✓					0.73	0.85	0.79
			✓				0.75	0.90	0.82
				✓			0.81	0.96	0.88
					✓		0.83	0.92	0.87
Ensemble KNN	✓					89.4	0.95	0.91	0.93
		✓					0.85	0.82	0.84
			✓				0.82	0.89	0.86
				✓			0.85	0.97	0.91
					✓		0.81	0.99	0.89

**Table 3:** Comparison of classifications results.

Ref#	Year	Results (%)
[75]	2018	0.66ACC
[74]	2019	0.69ACC
[63]	2020	0.74ACC
[76]	2020	Pre = 0.84, SE = 0.82 F1 = 0.83
[74]	2021	ACC = 0.73
[77]	2022	ACC = 0. 84, F1-score 0.84
**Proposed Method**		ACC = 90.6, Pre = 0.85 SE = 0.91, F1 = 0.88

**Table 4:** Configuration parameters of YOLOv2-ONNX model.

Classes	5
Anchors	13,17,18,21,43,49,73,108
Mini-batch size	64
Max epochs	100
Verbose frequency	30
Learning rate	0.001

**Table 5:** Localization results comparison.

Ref#	Year	Results
[78]	2022	0.95 IoU
**Proposed Method**		0.96 IoU, 0.98 mAP

## Data Availability

Data is downloaded from that P. Chen, “Knee osteoarthritis severity grading dataset,” Mendeley Data, v1 http://dx.doi.org/10.17632/56rmx5bjcr, vol. 1, 2018. https://radiopaedia.org/articles/osteoarthritis-of-the-knee (accessed on 5 July 2022).

## Nomenclature

G	Grid cells
d	Size of a bounding box
($a_{k}$,$b_{k}$), (${\hat{a}}_{k}$,${\hat{b}}_{k}$)	Center points of predicted and ground truth bounding box respectively
(${\sqrt{w}}_{k}$,${\sqrt{h}}_{k}$),(${\hat{\sqrt{w}}}_{k}$,${\hat{\sqrt{h}}}_{k}$)	Width, height
W	Weight
p	Probability
C	Conditional class
($s_{k}$,${\hat{s}}_{k}$)	The confidence score of predicted and ground truth bounding box
$\xi_{\left(P,R\right)}\left(i\right)$	LBP feature vector
$\xi_{\mathrm{fv}}$(i)	Fused feature vector
$\xi_{\mathrm{av}}$(i)	Feature vector of Alexnet
$\xi_{\mathrm{dv}}$(i)	The feature vector of Darknet-53
s	The operator that retains sign of differences
$X_{c}$	Center pixel value
$X_{k}$	Intensity values
P	Neighboring pixels
